# Opportunities for improving pLDH-based malaria diagnostic tests

**DOI:** 10.1186/1475-2875-10-213

**Published:** 2011-08-01

**Authors:** Robert C Piper, Ian Buchanan, Young Ho Choi, Michael T Makler

**Affiliations:** 1 Flow Incorporated. 6127 SW Corbett, Portland, OR 97239-3601, USA; 2 Vista Diagnostics, 11410 124th NE St., 484 Kirkland, WA 98034, USA; 3 Access Bio, Inc. 65 Clyde Rd. Suite A Somerset, NJ 0887, USA

## Abstract

**Background:**

Monoclonal antibodies to *Plasmodium *lactate dehydrogenase (pLDH) have been previously used to format immunochromatographic tests for the diagnosis of malaria. Using pLDH as an antigen has several advantages as a sensitive measure of the presence of parasites within patient blood samples. However, variable results in terms of specificity and sensitivity among different commercially available diagnostic kits have been reported and it has not been clear from these studies whether the performance of an individual test is due simply to how it is engineered or whether it is due to the biochemical nature of the pLDH-antibody reaction itself.

**Methods:**

A series of systematic studies to determine how various pLDH monoclonal antibodies work in combination was undertaken. Different combinations of anti-pLDH monoclonal antibodies were used in a rapid-test immunochromatographic assay format to determine parameters of sensitivity and specificity with regard to individual *Plasmodium *species.

**Results:**

Dramatic differences were found in both species specificity and overall sensitivity depending on which antibody is used on the immunochromatographic strip and which is used on the colorimetric colloidal-gold used for visual detection.

**Discussion:**

The results demonstrate the feasibility of different test formats for the detection and speciation of malarial infections. In addition, the data will enable the development of a universal rapid test algorithm that may potentially provide a cost-effective strategy to diagnose and manage patients in a wide range of clinical settings.

**Conclusion:**

These data emphasize that using different anti-pLDH antibody combinations offers a tractable way to optimize immunochromatographic pLDH tests.

## Background

Rapid tests for the diagnosis of malaria offer the potential to accurately detect and follow malaria infections in patients who live in remote areas without access to modern medical clinics. These tests use a wicking strip with immobilized antibodies to a particular antigen. Infected blood is wicked up the strip and the presence of the antibody-captured antigen is revealed with a coloured bead or colloidal gold, which is conjugated to a second antibody that also binds the antigen of interest [1,2].

Most currently commercially available malaria diagnostic rapid tests are based on the detection of either *Plasmodium falciparum *HRP2 protein or *Plasmodium *lactate dehydrogenase (pLDH) [3-5]. HRP2 tests are limited to the detection of *P. falciparum *while pLDH-based tests can detect multiple species of malaria parasites. The performance and potential benefit of such tests has been established for some time, but deploying such technology effectively remains a challenge. Scores of studies to evaluate the performance of malaria rapid diagnostic tests have been conducted [3-5]. While the general consensus has emerged that they perform well collectively, results have varied and have led investigators to a wide range of conclusions about the validity and utility of rapid tests. One of the largest potential causes of variability is the source of the rapid test itself. Currently, there are dozens of tests available from a variety of manufacturers. While efforts to systematically evaluate their quality have been instigated by WHO [6], more can be done to accurately define the performance characteristics and capabilities of these tests and to learn more about the behavior of the antigens that are targeted by these tests.

One advance would be to better understand how the targeted antigens are recognized by the antibodies used in the commercially available tests. For instance, a molecular characterization of HRP protein in *P. falciparum *has identified variability in the number and type of histidine-rich repeats within HRP2 [7,8]. In addition, histidine-rich repeats are found in other *P. falciparum *proteins that could potentially cross react with antibodies directed to HRP2. Establishing what type of histidine repeats are recognized by antibodies used in rapid tests and matching those data with test performance would be an important denominator to help provide accurate comparisons of test performance and predict their utility. Most commercially available HRP2 based tests rely on a single monoclonal antibody but can also incorporate polyclonal antibodies. So far, the empirical specificity of such reagents has not been systematically examined.

A panel of monoclonal antibodies directed to *Plasmodium*-LDH has been previously developed [1,9,10]. These antibodies show different specificities to the four species of malaria parasites that typically infect humans. Moreover, some of these antibodies have been incorporated into commercially available tests. It was found that differences in the combination of antibodies can have dramatic effects on the performance capabilities of a rapid test. These effects encompass changes in both specificity and sensitivity. Presented here is a thorough empirically derived matrix for how the pLDH antibodies perform in combination within the rapid diagnostic format. These results emphasize that variability among commercially available tests could come from different formulas of antibodies used and that with proper attention, optimizing this parameter may lead to better tests. In addition, these data support the proposal of new test formats that can sequentially diagnose malaria with high sensitivity and then uniquely identify which *Plasmodium *species is/are present within infected blood. This latter step might not only identify blood infected with individual species of *Plasmodium malariae*, *Plasmodium vivax*, *Plasmodium falciparum*, or *Plasmodium ovale *parasites but can also identify species of mixed infection.

## Methods

### Test strip construction

Test strips were made according to previous studies [1]. All antibodies were adjusted to 1 mg/ml in PBS prior to striping at 1 μl/cm. Antibodies were striped onto Millipore nitrocellulose membranes using a CAMAG striper (Muttenz, Switzerland).

### Running buffer

The buffer used to run the test strips and dilute the pLDH enzymes was 100 mM Na Borate (pH 8.), 1% Triton X-100, 1% Lactose, 1% Casein. Reagents were purchased from Sigma (St. Louis, MO), except for the Hammersten grade casein that was purchased from BDH (Poole, UK).

### Gold conjugates

Fresh solutions of trisodium citrate (1%) and Gold chloride (1%) were filtered through a 0.2 micron filter. One ml of gold chloride solution was added to purified water (100 mls) stirring in an acid washed 250 ml Erlynmeyer flask. The solution was brought to a slow boil and then added to it was 12 mls of the trisodium citrate solution. The solution was kept at a low boil until pink, after which 8.4 mls of the 1% gold choride solution was added to yield a dark purple gold colloid. The solution was maintained at a boil until the colloid matured to a purple-reddish color. Heat was removed and the colloidal solution stirred for an additional 15 min. The resulting solution yielded a colloidal gold measuring an optical density of 10 at 530 nm.

To conjugate the colloidal gold a 50 ml conical polypropylene tube was used to serially mix 2 mls of 0.1 Tris base pH 8.3, 2 mls of monoclonal antibody at 100 μg/ml previously dialyzed in 5 mM HEPES pH 6.5. 10 mls of colloidal gold solution was then added to the conical tube and mixed at 25°C for 5 min. 2.5 mls of Running Buffer lacking Triton X-100 was then added and mixed for an additional 5 min.

### Recombinant pLDH

Recombinant pLDH isoforms for *Plasmodium falciparum *(*Pf*-LDH), *Plasmodium malariae *(*Pm*-LDH), *Plasmodium ovale *(*Po*-LDH) and *Plasmodium vivax *(*Pv*-LDH) were made as previously described [1,9]. Crude lysates of *Escherichia coli *induced to express pLDH isoforms were used as the source of antigen. The sequence previously described for *Plasmodium knowlesi *LDH [10] was used to construct a synthetic gene engineered for expression of *Pk*-LDH in *Escherichia coli*. Enzyme activity was measured by the conversion of APAD and Lactate to APADH and Pyruvate as previously described [9,11,12]. Enzyme activity was adjusted to 100 μM -min - ml to make comparable lysates stocks, which were stored at -80°C in 5% glycerol in aliquots of 20 μls. For *P. falciparum *pLDH, this level of activity is roughly equal to a blood sample containing 5 × 10^7 ^parasites/μl or 1000% parasitaemia (10 parasites per red blood cell). This calculation is based on estimates from *in vitro *grown infected red blood cell cultures with an estimated 10% parasitaemia wherein it was found that such samples contained an average an activity of 1 μmol -min -ml. Previous biochemical studies on purified recombinant pLDH demonstrated that the all human *Plasmodium *species show a specific activity of ~1000 μmol -min-mg for the conversion of APAD to APADH in the presence of lactate [9,11], a value ~5 fold higher than conversion of NAD to NADH owing to the greater Kcat for the APAD coenzyme. Using this as a basis for calculation, the estimation method used here predicts that a 10 μl sample of infected whole blood at 1% parasitaemia (equivalent to 50,000 parasites/μl) will have 1 ng pLDH. This estimate is in line with independent studies based on ELISA assays using commercially available enzyme standards and cultured parasites where it was estimated that a 10 μl sample of 1% parasitaemia contains 0.3 ng pLDH [13].

### Immunochromatographic assay

Enzyme dilution samples (10 uls) were made in 96 well polystyrene plates. 2 drops of running buffer were added to each well as well as 10 μls of gold-antibody conjugate. Test strips were inserted into the wells and allowed to wick the sample for 10 min. Strips were then moved to a fresh well containing 4 drops of running buffer and allowed to clear for 10 min before being read. Strips were air dried prior to photography.

### Immunocapture pLDH assay

The immunocapture assay was based on previously described pLDH immunocapture assay [1]. Polystyrene ELISA plates were incubated with 200 uls of goat-anti-mouse affinity purified antibody at 0.1 mg/ml in PBS overnight. Wells were washed three times in PBS and then incubated with monoclonal antibody diluted in PBS containing 1 mg/ml BSA. Monoclonal antibodies were allowed to bind for 2 hrs. Wells were washed three times in PBS and then incubated with a solution of recombinant enzyme (diluted 1:20 from stock in running buffer) for 1 hr. Wells were then washed three times in PBS and reacted with 0.1 M Tris pH 9.0, 1 mM APAD, 100 mM Lactate 0.2% Triton X-100, 0.3 mM Nitroblue tetrazolium, 0.1 mg/ml Diaphorase and monitored spectrophotometrically for the production of formazan salt at 585 nm.

## Results

### Antibodies to pLDH

**Figure 1:**
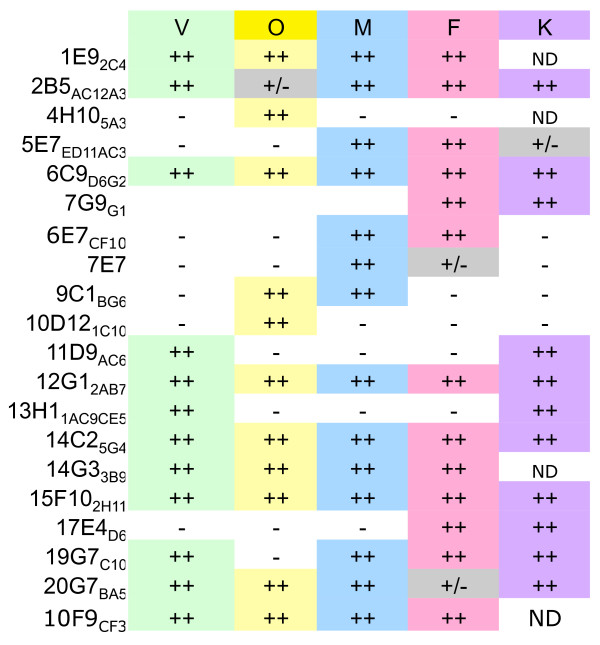
**Enzyme pLDH capture assay**. The indicated antibodies tested in an immune-capture ELISA format for the ability to immobilize pLDH. Captured enzyme activity was assessed by the conversion of lactate and NAD to pyruvate and NADH. Results were then graphically condensed to indicate none (-) strong (++), or very weak (-/+) captured enzyme activity.

Previous studies have characterized monoclonal antibodies to pLDH. These efforts were continued to produce a comprehensive panel of stable monoclonal cell lines that produce reliable antibodies that react strongly with pLDH without reacting with human LDHs [1]. Their specificity was first evaluated in an enzyme capture assay (Figure 1). Each monoclonal antibody was immobilized in an ELISA plate, and recombinant LDH was allowed to bind to the antibody. Unbound pLDH was washed away and bound pLDH was assayed by monitoring the conversion of Lactate and NAD to pyruvate and NADH. Importantly, each antibody was able to capture active pLDH demonstrating that the epitopes recognized were localized to the enzyme surface. These results also showed remarkable specificity with the ability of some antibodies to distinguish pLDH from different species of malaria. So called "pan" antibodies, which recognized all pLDH species, included 1E9, 6C9, 14C2, and 15F10. *Plasmodium falciparum*-specific antibodies included 7G9 and 17E4. Antibodies that exclusively captured *P. vivax *LDH included 11D9 and 13H1; antibodies reactive with only *P. ovale *were 4H10 and 10D12.

Other antibodies showed a mixed set of specificities. For instance, 19G7 reacted with all pLDH isoforms with the exception of *P. ovale*. This antibody was incorporated into the original OptiMAL assay, which utilized two capture lines [1]. One utilized 17E4, a *P. falciparum *specific antibody, and 19G7 to detect *P. falciparum *as well as additional species. This test was found not to recognize *P. ovale *infections [14] as confirmed here by the inability of the 19G7 to recognize recombinant *P. ovale *LDH in the capture assay. The specificity profile of 19G7 within the capture assay is shared by the additional antibody 2B5. The antibody panel also has antibodies that bind to both *P. malariae *and *P. falciparum *LDH but do not bind *P. vivax *or *P. ovale *LDH. These include antibodies 7E7 and 6E7. Additional experiments showed the of reactivity of the 7E7 antibody to be much stronger for *P. malariae *than for *P. falciparum *LDH which could be potentially exploited by using different buffer conditions to bias reactivity enough to make 7E7 a *P. malariae*-specific antibody. While the ratio of reactivity of 7E7 to *P. malariae *LDH and *P. falciparum *LDH could be increased in exploratory studies by adjusting constituents in the buffer, a set of assay conditions that excluded all reactivity of 7E7 to *P. falciparum *LDH could not be found. Thus, no antibody was found that had exclusive specificity towards *P. malariae *LDH.

Other antibodies showed different repertoires of reactivity. For instance, 20G7 reacts strongly with *P. malariae*, *P. ovale*, and *P. vivax *but only very weakly with *P. falciparum *LDH, theoretically making it possible for this antibody to differentiate all non-*falciparum *infections if optimized correctly. The 6E7 and 9C1 antibodies were strongly reactive towards *P. malariae *LDH, the former also reacting with *P. falciparum *LDH and the latter also reacting with *P. ovale *LDH. In principle, the reaction profile of 6E7 and 9C1 might provide the tools to uniquely detect *P. malariae *infections. Although both antibodies cross react with other pLDH species, the only species they both bind is *P. malariae*. Thus a rapid test with a capture line of 6E7 and a reporter bead coated with 9C1 theoretically would form a positive reaction in the presence of *P. malariae *LDH.

### Placement of epitopes

**Figure 2:**
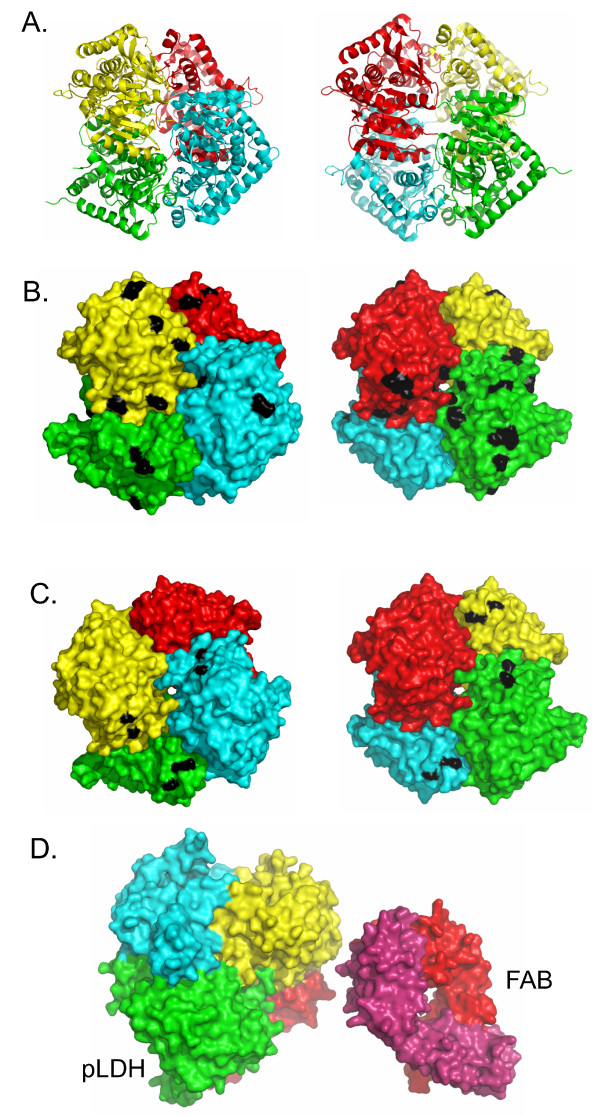
**Hypothetical epitope surface of pLDH**. Structures of *Pf*-LDH and *Pv*-LDH or predicted structures of *Po*-LDH and *Pm*-LDH determined by molecular threading are shown in a computed chimeric pLDH tetramer where each of the 4 subunits is taken from the four human malarias. *Pf*-LDH, red; *Pv*-LDH, green; *Pm*-LDH, cyan, *Po*-LDH, yellow. **A**. Shows ribbon structure of the pLDH tetramer. **B**. Shows the molecular surface of the pLDH tetramer with surface-exposed residues unique to the different pLDH isoforms respectively indicated in black. **C**. Shows the molecular surface and residues shared between *Po-*, *Pm-*, *Pv*-LDH but that are different from *Pf*-LDH are indicated in black. The indicated residues are candidates for the epitopes of 9C1 and 20G1 antibody. **D**. Shows the pLDH tetramer with the heavy (red) and light (purple) chains of a typical antibody FAB fragment. This is to show the relative scale of the molecules to demonstrate that an epitope could conceivably span patches found in two different pLDH subunits.

Two pLDH structures from human malarias have been solved by X-ray crystallography [15,16]. Both are of the native tetrameric form of the enzyme and highly similar. These structures were used to map which surface exposed residues might account for the species differences in antibody reactivity (Figure 2). Surprisingly, these differences were scattered across the surface of pLDH and did not focus to large discrete patches. Thus, the epitope differences that determine differential binding of various antibodies are likely to be small, perhaps 1-2 amino acid differences as determinants of species specificity.

### Rapid test combinations

Next examined was how the antibodies performed in an immunochromatographic test format. These tests have two components: one is the nitrocellulose strip that has an antibody solution stripped across it to make a band where pLDH can be immobilized; the other is a colloidal gold antibody conjugate which is immobilized on the strip in the presence of a bridging antigen. Successful capture of the gold conjugate on the antibody stripe requires that the two antibodies used in the test bind to different non-competing epitopes.

**Figure 3:**
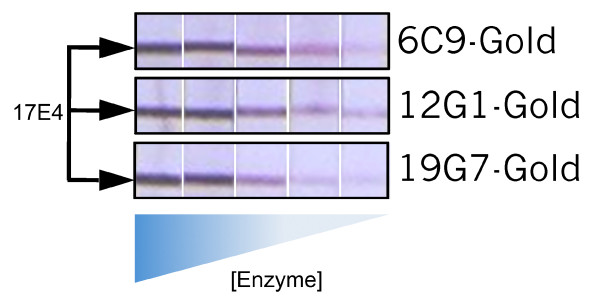
**Sensitivity of detecting *P. falciparum *LDH as a function of antibody gold conjugate**. Test strips with immobilized 17E4 antibody specific for *Pf*-LDH were used to test for reaction to *Pf*-LDH. The dilution series (1:10) started with a pLDH solution diluted 1:100 from the stock solution to produce a sample solution estimated to represent a 10% parasitaemia. Tests were visualized with colloidal gold conjugated to the indicated antibodies.

It was first tested whether antibody on the gold conjugate made a difference with regard to sensitivity (Figure 3). Test strips were made with the *P. falciparum*-specific antibody 17E4 and reacted in the presence of recombinant *P. falciparum*-LDH in combination with colloidal gold conjugates made with 6C9, 12G1, or 19G7 antibodies. The antibodies used in the gold conjugate all react strongly with *Pf*-LDH in the antibody capture assay (Figure 1), yet differences were found with regard to their sensitivity when used on the colloidal gold. Namely, the 6C9-gold and the 12G1-gold gave stronger signals with lower amounts of pLDH than did the 19G7 gold conjugate. These results indicate that sensitivity of a particular malaria rapid test format might be optimized by using different antibody colloids.

**Figure 4:**
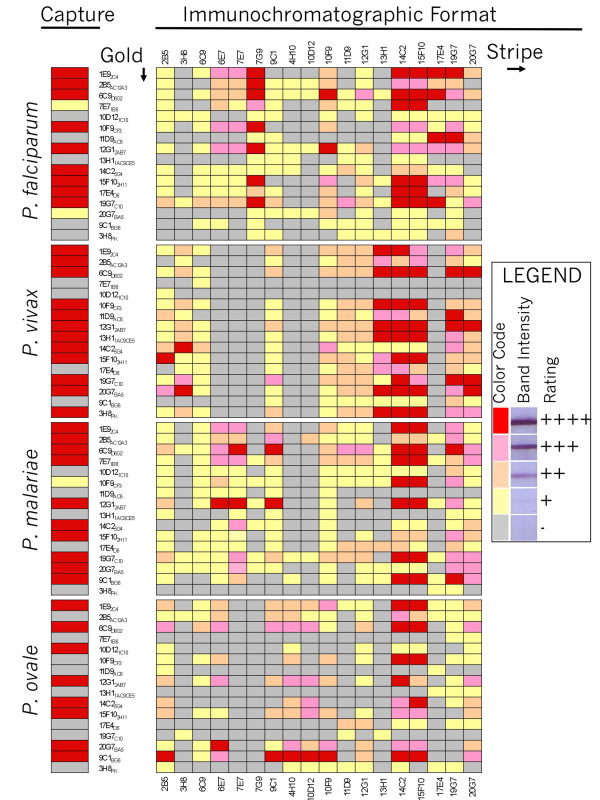
**Rapid Test Reaction Matrix**. Left: Reactivity of various species of pLDH in the enzyme capture assay is shown for comparison. Right: The antibodies indicated in the Y-axis were conjugated to colloidal gold under standard conditions and used to test for reactivity with recombinant pLDH of *P.falciparum, P. vivax, P. malariae*, and *P. ovale*. Each enzyme conjugate was used with a test strip containing the indicated antibodies (X-axis) immobilized on the test strip. Line intensity of each reaction was color coded as a function of its intensity according the scheme indicated in the inset legend. The pLDH enzyme solution mimicked a sample of 1% parasitaemia.

A comprehensive examination was then conducted to determine how each antibody combination would work in the immunochromatographic format (Figure 4). A colloidal gold antibody conjugate for each of the antibodies was created. Importantly, a standard set of conditions was used for making these conjugates to facilitate comparison. However, it is known that differences in buffer conditions during the conjugation procedure can have effects on the quality of the resulting conjugate. Thus, each of the antibody gold conjugates used in this study still has the potential of being further optimized. All of these gold conjugates were evaluated using strips in which each antibody was striped. Recombinant pLDH from *P. falciparum *(Pf), *P. malariae *(Pm), *P. ovale *(Po), and *P. vivax *(Pv) was used for each test using a standard running and wash buffer. The enzyme was diluted in running buffer to match the concentration of enzyme estimated to be in a typical blood sample with a parasitaemia of 1%. This value was chosen as generally low enough to indicate relative sensitivities of antibody combinations based on the intensity of the reaction band and high enough to demonstrate the fidelity of the specificity for individual pLDH species isoforms.

In general, the antibody specificities determined in the pLDH capture assays (Figure 1) were recapitulated in the rapid test format (Figure 4). However, the relative strength of the reaction line varied among the combinations. Thus, some antibodies on the capture line were well suited to particular partner conjugate antibodies but not others. One antibody that worked very well in combination with the *P. falciparum *and *P. vivax *specific antibodies was 6C9, which to the knowledge of the authors, is routinely conjugated to the colloidal gold reporter in currently available commercial rapid malaria pLDH tests.

The matrix also tested how well a single antibody performed when it was used on both the capture line and reporter gold conjugate. In principle, this type of approach should still allow the antigen to form a sandwich between the capture line and the reporter conjugate since pLDH is a tetramer and there could be four epitopes per pLDH to allow for crosslinking. In some cases, this approach worked well. For instance, the pan-specific antibody 15F10 gave a robust signal for all malaria species when used as both the capture and conjugate. For most antibodies, however, this approach yielded only weak signals, indicating that competition between the capture antibody and the conjugate antibody for the epitopes available on pLDH was formidable. This can be seen with antibodies such as 6C9 and 19G7. These are capable of yielding strong signals with *P. falciparum*, *P. vivax*, or *P. malariae *LDH when used on either the capture or conjugate; however, when used for both only a meager signal was achieved (Figure 4).

There were many instances where the performance of a particular antibody combination differed from what would be predicted from initial characterization by the immuno-capture assay. For example, when the *P. falciparum*-specific antibody 17E4 was conjugated to gold, it could now react with *P. vivax *LDH immobilized by the 13H1 antibody and *P. malariae *LDH immobilized by the 9C1 antibody. Similarly, when 11D9 and 20G7 were used for the conjugate and stripe respectively, they detected Pf-LDH even though neither of those antibodies binds *Pf*-LDH by the enzyme capture assay nor do they bind *Pf*-LDH with matched with other antibodies. Importantly, when used as the capture antibody in combination with the 6C9-conjugated gold, the 17E4, 13H1, 9C1, 11D9 and 20G7 antibodies all showed the specificity profile that they had in the immunocapture assay, suggesting that the antibodies may react differently depending on the surface (test strip or gold conjugate) to which they are immobilized. A similar characteristic was found for the pan-specific antibodies 14C2 and 15F10. When they were immobilized on the strip, they reacted strongly with a number of gold-conjugated antibodies for all species of pLDH, and fit the predictions from the immuno-capture assay. However, when conjugated to gold, 14C2 and 15F10 had extremely limited reactivity both in terms of the repertoire of antibodies on the test strip they could sandwich with as well as the species of pLDH they could react against. These data emphasized that the rapid test formulation could not be completely predicted from the capture data alone.

### Specific detection of *P. falciparum*

**Figure 5:**
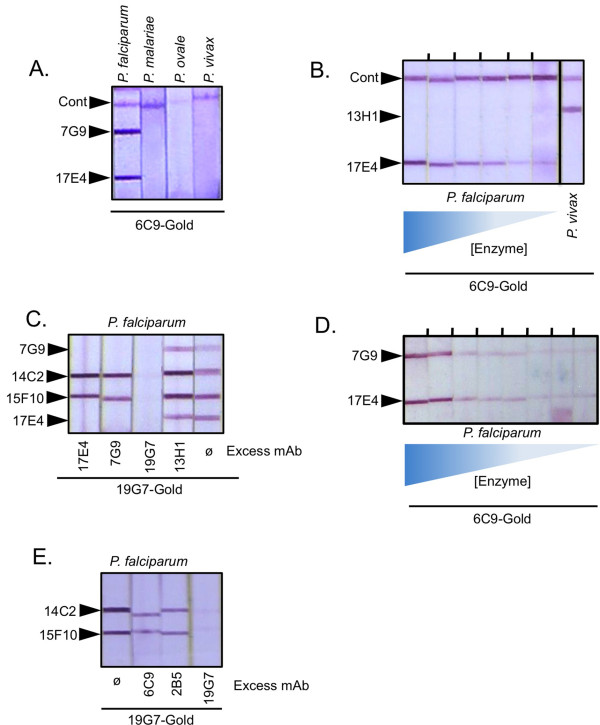
**Specificity of epitopes**. A. Test strips with immobilized 7G9 and 17E4 were reacted with high levels of pLDH from the indicated species (1:10 dilution of stock enzyme to mimic a > 100% parasitaemia). Immunoreactive bands were visualized with 6C9-conjugated colloidal gold. A control (Cont) line comprised of goat anti-mouse antibody is also shown. B. Test strips with 13H1 and 17E4 were reacted with a serial dilution (1:10) of *Pf*-LDH where the starting concentration represented a > 100% parasitaemia. C. Competition experiment to demonstrate epitope differences and similarities. Prior to wicking up the strip, the *Pf*-LDH sample was incubated with buffer (ø) or buffer containing excess 17E4, 7G9, 19G7, or 13H1 antibodies as indicated at a final concentration of 200 μg/ml for 2 min. D. A serial dilution (1:10) titration of *Pf*-LDH was analyzed on test strips with immobilized 7G9 and 17E4. Bands were visualized with 6C9-conjugated colloidal gold. E. Test strips with immobilized 14C2 and 15F10 were reacted with *Pf*-LDH and 19G7-conjugated colloidal gold. The *Pf*-LDH samples were preincubated with buffer alone (ø) or free 6C9, 2B5, or 19G7 antibody for 2 min prior to analysis.

Two antibodies (17E4 an 7G9) showed excellent specificity detecting *Pf*-LDH while not binding *Pm*-LDH, *Po*-LDH, or *Pv*-LDH (Figure 5A). The experiment shown used high concentrations of pLDH, levels that would exceed that in a theoretical patient sample of 100% parasitaemia. Figure 5B shows that a range of *Pf*-LDH concentrations, starting at levels in excess of that estimated in a sample of 100% parasitaemia, reacted strongly with 17E4 antibody but did not react at all with the *P. vivax*-specific 13H1 antibody. These results demonstrate high fidelity of particular antibodies for particular isoform species of pLDH and diminish the likelihood that antigen cross-reactivity explains observations of non-specificity between *Pf *and *Pv *reported for some patient samples analyzed by commercial malaria rapid tests that incorporate 13H1 or 11D9 antibodies [17].

Of the *Pf*-specific 17E4 and 7G9 antibodies, many of the earlier versions of pLDH tests used in clinical studies incorporated 17E4. The high specificity of these antibodies is restricted to their use as the immobilizing antibody on the test strip; as noted above some loss of specificity was observed when 17E4 was used instead on the gold conjugate (Figure 4). Overall, both 17E4 and 7G9 have similar characteristics. Indeed, the epitopes for 7G9 and 17E4 largely overlap as determined by competition experiments (Figure 5C). Here, both 7G9 and 17E4 as well as two pan-specific antibodies 14C2 and 15F10 were stripped onto the nitrocellulose strips. These strips were then reacted with *Pf*-LDH and 19G7-conjugated gold in the absence and presence of excess unbound antibodies. In the absence of excess competing antibody all four striped antibodies immobilized *Pf*-LDH and the gold conjugate as expected. In the presence of excess 19G7 all signals were inhibited because the free 19G7 bound its epitope on *Pf*-LDH and effectively competed with the 19G7 on the gold conjugate. No competition was observed with excess 13H1, which is specific for *Pv*-LDH and thus does not bind to the *Pf*-LDH. Importantly, excess 17E4 effectively competed away both the 17E4 band as well as the 7G9 band. Similarly, excess 7G9 competed away both the 7G9 band and the 17E4 band, indicating that 7G9 and 17E4 compete for a similar epitope on the surface of *Pf*-LDH. Both antibodies also show comparable sensitivity. This was revealed in a titration series (Figure 5D), where *Pf*-LDH was serially diluted and tested against strips with both 17E4 and 7G9 using the pan-specific 6C9-gold conjugate. These experiments revealed both antibodies gave similar band intensities across a broad concentration of *Pf*-LDH samples. Although 17E4 and 7G9 have similar performance overall, there are subtle differences between these two antibodies. This is important because those subtle differences might have large effects with regard to optimizing a final commercial rapid malaria test. For instance, when 7G9 and 17E4 are used on the test strip, the 7G9 stripe formed a much more intense band than 17E4 when used against gold conjugated to 2B5 or 10F9 (Figure 4). Also, the unexpected reaction of *Pf*-LDH with 11D9-conjugated gold (ostensibly *Pv*-specific) and immobilized 17E4 was not observed with immobilized 7G9 in combination with 11D9-gold (Figure 4).

### Pan-specific antibodies

Antibodies that recognize all species of pLDH are important components for a pLDH rapid test. Earlier versions of pLDH-based tests used two "pan" specific antibodies. The 6C9 antibody was used as the gold conjugate and the experiments here confirm that this configuration performs very well in terms of both sensitivity and specificity. The 19G7 antibody used in older tests was initially used as the immobilized pan specific antibody that would form a band with both *Pf*-LDH and other pLDH isoforms. Subsequent studies, however, showed that the 19G7 antibody does not recognize *Po*-LDH. The expanded panel of monoclonal antibodies reveals additional pan antibodies. In the pLDH enzyme capture assay, 14C2 and 15F10 recognize all forms of pLDH and thus are comparable to 6C9; the 2B5 antibody behaves like 19G7 in that it captures *Pf*-, *Pm*-, and *Pv*-pLDH isoforms but fails to robustly capture *Po*-LDH. Experiments to determined how distinct the corresponding epitopes for these antibodies were conducted using the competition assay described above (Figure 5E). Here it was found that 2B5 does not compete with 19G7, 14C2, or 15F10 suggesting that 2B5 indeed has a different epitope than the 19G7 and the others. Also, it was found that 6C9, which robustly binds all species of pLDH does not compete with 14C2 and 15F10, which also bind all species of pLDH. Thus, these results have identified two pairs of true pan-specific antibodies (6C9:14C2 and 6C9:15F10) that complement each other -rather than compete--for pLDH binding. This is important since it may now be possible to make a single test line capable of detecting all species of human malaria parasites. Such a test would be expected to be quite sensitive since it uses two non-competing antibodies on the test line and gold conjugate respectively, thus avoiding the inhibitory competition effect observed when a pan-specific antibody is used on both the test strip and gold conjugate. Figure 4 supports this prediction as these combinations yield some of the most intense bands found within the systematic matrix comparison.

### Differentiation of four human malarial parasites

**Figure 6:**
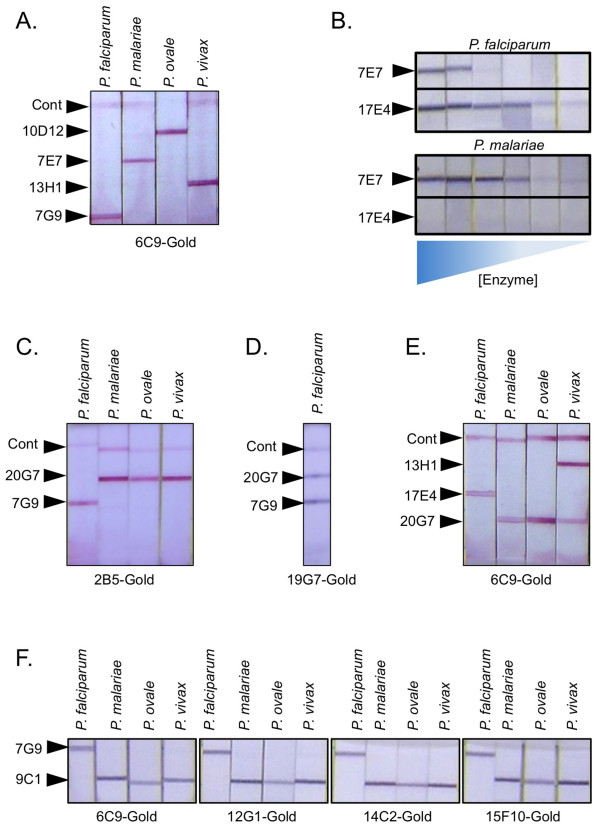
**Differentiating pLDH orthologs**. A. Test strips with the indicated immobilized antibodies were reacted with recombinant *Pf*-LDH, *Pm*-LDH, *Po*-LDH or *Pv*-LDH and visualized with 6C9-conjugated colloidal gold. A goat anti-mouse line is also indicated (Cont). A 1:100 dilution of enzyme (~ 10% parasitaemia) was used. B. A dilution series (1:10) of *Pf*-LDH and *Pm*-LDH was reacted with test strip containing immobilized 7E7 and 17E4 antibodies. The starting concentration of enzyme approximates a blood sample of 10% parasitaemia. C. Example of a *Pf*/non-*Pf *test format that can detect all four species of human malaria and distinguish *Pf*-LDH from the other species. Test strips contained immobilized 20G7 and 7G9 antibodies as well as a control (Cont) goat anti-mouse line. Reactions were visualized with gold conjugated to 2B5. A 1:100 dilution of the enzyme (~10% parasitaemia) stocks was used for the samples. D. The same 20G7-7G9 strip used in C was used to detect *Pf*-LDH using 19G7-conjugated colloidal gold. E. Similar assay format as shown in C except with the addition of an immobilized stripe of *Pv*-specific 13H1 antibody. F. Alternative *Pf*/non-*Pf *test format. Test strips containing immobilized 7G9 and 9C1 were reacted with pLDH from the indicated species. Reaction bands were visualized with colloidal gold conjugated to either 6C9, 12G1, 14C2 or 15F10 as indicated.

The matrix of antibody combinations (Figure 4) revealed several combinations with selectivity for different malaria species. This allowed us to format a number of test prototypes that combine high sensitivity with the ability to discriminate different species. Figure 6A shows the ability to detect and differentiate all four species of human malaria on a single test strip. Here *Pf*-LDH is revealed with immobilized 7G9, *Pm*-LDH is revealed with immobilized 7E7, *Po*-LDH by 10D12, and *Pv*-LDH by 13H1. A 6C9 conjugated to gold was used for detection, which nicely yielded unique single lines for each pLDH isoform. The 7E7 antibody was used as the *Pm*-specific antibody; however, 7E7 can react at higher concentrations of *Pf*-LDH (Figure 6B). Thus, while most *P. falciparum *infections are likely low enough not to cause this cross-reactivity, a very high *Pf *parasitaemia may give a false-positive *Pm *reaction in such a format. Even with this limitation, optimization of the gold conjugate, running buffer, and other parameters may eliminate this complication in a finished test. Alternatively, a set of strips using different gold conjugate antibodies could be used to avoid this cross-reactivity (see below).

The experiments also showed that the 20G7 antibody did not react or reacted extremely poorly with *Pf*-LDH while giving strong reactions with *Pm*-, *Pv *-, and *Po*-LDH. By co-striping 20G7 with 7G9, a test was made that could differentiate *P. falciparum*-LDH from non-*P. falciparum *LDH. Potentially, this format would be quite useful for making the major differential diagnosis required in the determination of malarial infection. Moreover, it has an advantage over earlier pLDH based tests that contain a *Pf*-specific and pan-specific line since the *Pf*/non-*Pf *test (Figure 6C) can identify mixed infections (eg *Pf *and *Pv*) while the *Pf*/pan test cannot. Careful attention has to be paid to the type of antibody combinations used for this format. For instance, a test strip with 7G9 and 20G7 works well as a *Pf*/non-*Pf *test when used with 2B5-conjugated gold (Figure 6C). However, when the same test strip is used with 19G7-conjugated gold, the species selectivity is lost since 20G7 can now react with *Pf*-LDH bound to 19G7-conjugated gold. It is not clear how there is an apparent "gain" in binding activity of the 20G7 antibody under these conditions. However, it is likely that subtle changes in the surface or conformation of pLDH that result from binding one antibody may inhibit or promote binding of a second antibody via a weak allosteric effect.

Figure 6E takes the Pf/non-*Pf *test a step further by adding a 13H1 stripe to the test format. This format could further confirm that the non-*Pf *species is *P. vivax *but comes with the caveat that the test cannot distinguish a mixed *Pv/Pm *or *Pv/Po *infections. Such mixed infection may be too rare to confound the effective use of such a test however.

The antibody matrix also revealed an alternative combination for making a *Pf*/non-*Pf *test using 7G9 and 9C1, respectively on the test strip (Figure 6F). This format reveals *Pf*-LDH reacts only on the 7G9 line; *Pm*-, *Po*-, and *Pv*-LDH are only revealed on the 9C1 line. Furthermore, this immunochromatographic strip works well with a number of different antibodies conjugated to gold including the 6C9 gold conjugate. The *Pf*/non-*Pf *format using 7G9 and 9C1 was very strong and specific for *Pf*, *Pm*, and *Pv*; however, *Po *reactivity was slightly less than ideal. One way to optimize this reaction would be to supplement the 20G7 line by mixing in the *Po*-specific 10D12 or 4H10 antibody prior to striping to make a mixed antibody line with stronger reactivity to *Po*-LDH.

### Specific detection of *Plasmodium knowlesi*

**Figure 7:**
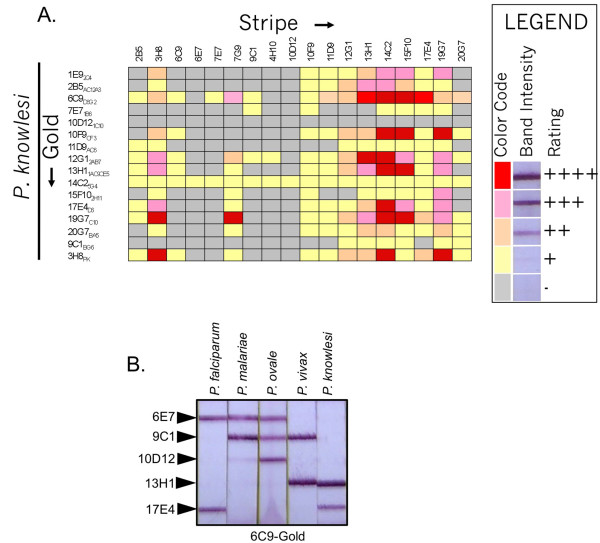
**Differentiating *P. knowlesi***. A. Recombinant *Pk*-LDH was used in a immunochromatographic format matrix. The pLDH enzyme solution was diluted to mimic a sample of 1% parasitaemia. B. Test strips with the indicated immobilized antibodies were reacted with *Pf*-LDH, *Pm*-LDH, *Po*-LDH, *Pv*-LDH or *Pk*-LDH and 6C9-conjugated gold. The pLDH enzyme mimicked a sample of 10% parasitaemia.

*Plasmodium knowlesi *(*Pk*) is ostensibly a primate malaria. However, it can infect humans and cause a high level of pathology due to its ability to create large parasite burdens [18]. Earlier studies showed that *Pk*-LDH binds to both the *Pf*-LDH specific antibodies 7G9 and 17E4 and also the *Pv*-specific antibodies 11D9 and 13H1 [10]. In an effort to find tools to discriminate *Pk*-LDH from the four canonical human pLDH isoforms, various combinations of antibodies for their ability to react with *Pk*-LDH in the immunochromatographic assay was assessed (Figure 7). These results confirmed that *Pk*-LDH binds both 17E4 and 13H1 and additionally revealed that *Pk*-LDH does not bind the 9C1 antibody (which does bind *Po*, *Pv*, and *Pm *LDH isoforms). Also, *Pk*-LDH did not bind the 6E7 antibody, which is able to bind *Pf*, *Pm*, and *Po*-LDH. Thus, the discriminatory power of using the 9C1 antibody in combination with others would enable the design of a test format that could better discriminate a *P. knowlesi *infection from a *P. vivax *or *P. falciparum *infection. Humans infected with *P. knowlesi *are typically misdiagnosed with *P. malariae *when microscopy is used. Since the pLDH reaction pattern of *Pk*-LDH is very different from that of *Pm*-LDH (Figure 7B) the combined use of both RDTs and microscopy would give a definitive differentiation between *P. malariae *and *P. knowlesi *infection.

## Discussion

It is clear from these studies that one pathway to optimize pLDH diagnostic tests is to explore the use of alternative antibodies that have slightly different binding characteristics and which make use of the subtle antigenic differences on the surface of the pLDH enzyme. Importantly, this is only one aspect for how to optimize a finished commercially viable test since issues of stability are also very important and formulations to achieve optimal stability may differ for different antibodies. In addition, the performance of the antibody combinations used here could potentially be enhanced by optimizing buffer conditions and solid support matrices used in the test strip, as well as conditions for making the reporter gold conjugates. The differences observed amongst the different monoclonal antibodies demand a precise approach to tailoring reagents, antibodies, and formats in order to achieve consistent and robust test kits. This may be best achieved by routine use of monoclonal antibodies that have consistent reactivity characteristics rather than by using polyclonal antibody mixtures that yield a variable mix of antibody specificities that may hamper reproducibility [19,20].

By mixing different monoclonal antibodies to pLDH, a number of effective rapid malaria test formats could be developed to meet the specific needs of particular clinical settings. This strategy also provides means to enhance the sensitivity and specificity of pLDH tests over existing rapid test formats. Combining several incremental improvements based on the observations presented here may be enough to evolve a new series of rapid tests that can meet a broad set of patient needs. Given the success so far in the performance of some pLDH tests, this pathway to improvement may make widespread effective and commercially sustainable diagnostics a reality in a relatively short time. Hence an extended search for more exotic biomarkers that face a long period of experimental validation before being employed in clinical practice as previously proposed [21] may not be needed if minor improvements are made to existing technology.

**Figure 8:**
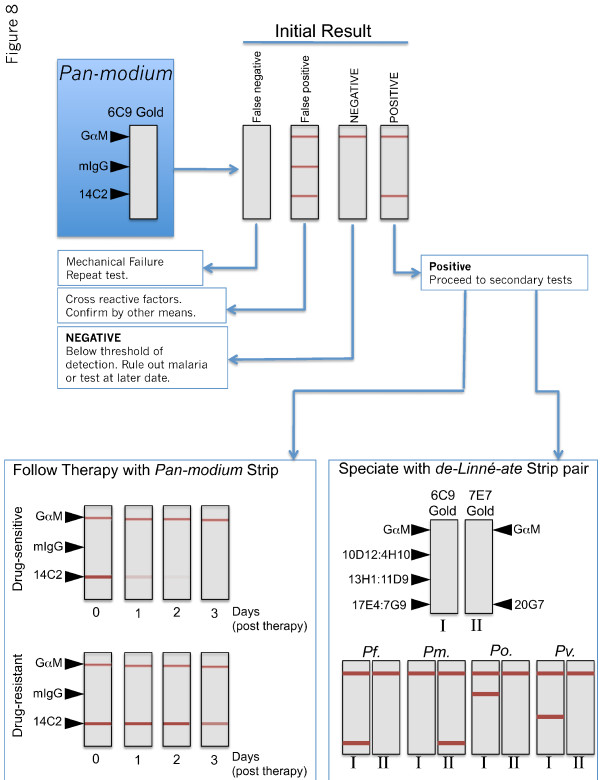
**Proposed diagnostic algorithm**. Model for how two different new test formats could be integrated into a uniform diagnostic regimen. Initial diagnosis would be made using a pan-specific test termed *Pan-modium*. If the test were positive, a second test to determine the species of malaria would be performed. This test, termed *de-Linné-ate *is comprised of 2 test strips (I and II) run in parallel. The *de-Linné-ate *strip contains the indicated antibodies and uniquely identifies each of the four human malaria parasite pLDH orthologs. Once an anti-malarial drug therapy has been initiated, progress towards clearing the malaria infection can followed using the all purpose *Pan-modium *strip.

From the characterization of the antibody combinations used here, a model is proposed for how new test formats could be developed and eventually integrated into a diagnostic algorithm that may be suitable for a wide range of clinical conditions that span the whole gamut of malaria prevalence (Figure 8). One format, dubbed "*Pan-modium*", contains a single line to indicate the presence of malaria parasites in a blood sample and uses two non-competing pan-specific antibodies. *Pan-modium *would be comprised of 14C2 or 15F10 antibody immobilized on the test strip and revealed by 6C9 conjugated gold. The *Pan-modium *strip would also contain two control lines. One line would be an anti-mouse IgG line, which would bind the gold-conjugate and serve as a control to make sure the test reagents were correctly wicked up the strip. This type of control line serves as the industry standard for immunochromatographic tests. The *Pan-modium *strip would also contain an additional line and be comprised of normal mouse IgG (mIgG). The mIgG line would not react with pLDH but would react with anything in patient blood that could non-specifically link mouse antibodies together. Such non-specific activity has been noted before and can cause apparent false-positives in pLDH tests [3-5,22]. By including the mIgG line, the user would have the ability to identify samples that have factors that cause such false-positive reactions and allow the user to use alternative means or buffer additives to obtain a diagnosis.

Once a patient is diagnosed with a viable parasitaemia in their blood with the *Pan-modium *strip, the clinician could then use a different test termed "*de-Linné-ate*", dubbed in homage to the taxonomous Carl Linné (Linneaus). The *de-Linné-ate *test would be formatted as a pair of test strips (I and II). One would be striped with the *Po*-specific antibodies 10D12 or 4H10; the *Pv*-specific 13H1 or 11D9; and the *Pf*-specific 17E4 or 7G9 antibodies. When used in conjunction with 6C9 gold, this test strip would uniquely identify *Po*, *Pv*, or *Pf *infections or mixed infections of those species. A second (II) test strip run in parallel would consist of 20G7 on the test strip and 7E7 conjugated to gold. This combination only reacts with *Pm*-LDH. By pairing 20G7 with 7E7, it avoids the slight cross reactivity that 7E7 has with *Pf*-LDH. This is because 20G7 does not readily bind *Pf*-LDH. Thus, the only pLDH isoform to bind both 20G7 and 7E7 (and thus produce a line) is *Pm*-LDH. Together, the pair of *de-Linné-ate *test strips, which could be multiplexed together in a single device, would give unambiguous identification of any and all species of malaria parasites in an infected sample. The addition the test could further be refined to also differentially diagnose *Pk*. This could be done by reconfiguring the *de-Linné-ate*-II strip to have immobilized 7E7 (a *Pm *line) and 9C1 (a *Po*, *Pm*, *Pv*) line and reacted with 20G7 gold. Here the *de-Linné-ate*-I strip would show both a "*Pv*" and "*Pf*" line but fail to give a 9C1 line in the *de-Linné-ate*-II strip thus indicating a *Pk *infection rather than a *Pf/Pv *mixed infection. In practice, however, the addition of 9C1 into a five-test-line combination to differentiate five species of malaria parasites may produce unneeded complexity for most clinical situations and cause confusion.

Once the species of malaria has been correctly determined, an appropriate drug therapy can be chosen. To follow the success of such therapy the clinician can then follow up with repeated analysis using the *Pan-modium *strip. Because pLDH levels closely correlate with viable parasitaemia [1,23], successful drug therapy results in loss of pLDH-test reactivity. If therapy failed to reduce reactivity on the *Pan-modium *strip, the clinician could confidently suspect a drug-resistant infection and change drug-therapy in response. In addition, because the *Pan-modium *uses a single anti-malaria antibody test line and could be adapted to a wide range of clinical conditions, the potential of a widely available, very low cost *Pan-modium *test that could be economically produced on a massive scale would make both the initial diagnosis and follow-up very affordable and effective.

## Conclusions

A panel of monoclonal antibodies against *Plasmodium *lactate dehydrogenase can be used in various combinations to uniquely identify all species of malaria parasites that infect humans. In addition, different combinations of antibodies were found to produce different levels of sensitivity within an immunochromatographic rapid test format. These results should help the development of new diagnostic test kits with greater specificity, sensitivity and ability to differentiate among malaria parasite species.

## List of abbreviations

pLDH: *Plasmodium *Lactate Dehydrogenase; *Pf*: *Plasmodium falciparum*; *Pm*: *Plasmodium malariae*; *Po*: *Plasmodium ovale*; *Pv*: *Plasmodium vivax*; *Pk*: *Plasmodium knowlesi*; HRP2: Histidine Rich Protein 2.

## Competing interests

RCP and MTM have developed the antibody panel for research purposes and for us in commercially available malaria diagnostic assays. IB and YHC are test developers and manufacturers that currently make and develop pLDH tests for sale. Antibodies are available for purchase from YHC and Access Bio.

## Authors' contributions

RCP helped design the experiments and wrote the manuscript. MTM designed and executed the experiments and edited the manuscript. IB refined experimental design, supplied key reagents, and edited the manuscript. YHC refined experimental design, supplied key reagents, and edited the manuscript. All authors read and approved the final manuscript

## References

[B1] Piper R Lebras J Wentworth L Hunt-Cooke A Houze S Chiodini P Makler M Immunocapture diagnostic assays for malaria using *Plasmodium *lactate dehydrogenase (pLDH) Am J Trop Med Hyg 1999 60 109 118 9988333 10.4269/ajtmh.1999.60.109

[B2] Shiff CJ Premji Z Minjas JN The rapid manual ParaSight-F test. A new diagnostic tool for *Plasmodium falciparum *infection Trans R Soc Trop Med Hyg 1993 87 646 648 10.1016/0035-9203(93)90273-S 8296363

[B3] Makler MT Piper RC Rapid malaria tests: where do we go after 20 years? Am J Trop Med Hyg 2009 81 921 926 10.4269/ajtmh.2009.09-0202 19996417

[B4] Wongsrichanalai C Barcus MJ Muth S Sutamihardja A Wernsdorfer WH A review of malaria diagnostic tools: microscopy and rapid diagnostic test (RDT) Am J Trop Med Hyg 2007 77 119 127 18165483

[B5] Murray CK Bennett JW Rapid diagnosis of malaria Interdiscip Perspect Infect Dis 2009 2009 415953 19547702 10.1155/2009/415953 PMC2696022

[B6] WHO Malaria Rapid Diagnostic Test Performance: Results of WHO product testing of malaria RDTs: Round 2 2009 World Health Organization http://www.wpro.who.int/internet/files/rdt/RDTMalariaRd2_FINAL.pdf

[B7] Baker J Ho MF Pelecanos A Gatton M Chen N Abdullah S Albertini A Ariey F Barnwell J Bell D Cunningham J Djalle D Echeverry DF Gamboa D Hii J Kyaw MP Luchavez J Membi C Menard D Murillo C Nhem S Ogutu B Onyor P Oyibo W Wang SQ McCarthy J Cheng Q Global sequence variation in the histidine-rich proteins 2 and 3 of *Plasmodium falciparum*: implications for the performance of malaria rapid diagnostic tests Malar J 2010 9 129 10.1186/1475-2875-9-129 20470441 PMC2893195

[B8] Baker J McCarthy J Gatton M Kyle DE Belizario V Luchavez J Bell D Cheng Q Genetic diversity of *Plasmodium falciparum *histidine-rich protein 2 (PfHRP2) and its effect on the performance of PfHRP2-based rapid diagnostic tests J Infect Dis 2005 192 870 877 10.1086/432010 16088837

[B9] Brown WM Yowell CA Hoard A Vander Jagt TA Hunsaker LA Deck LM Royer RE Piper RC Dame JB Makler MT Vander Jagt DL Comparative structural analysis and kinetic properties of lactate dehydrogenases from the four species of human malarial parasites Biochemistry 2004 43 6219 6229 10.1021/bi049892w 15147206

[B10] McCutchan TF Piper RC Makler MT Use of malaria rapid diagnostic test to identify *Plasmodium knowlesi *infection Emerg Infect Dis 2008 14 1750 1752 18976561 10.3201/eid1411.080840 PMC2630758

[B11] Deck LM Royer RE Chamblee BB Hernandez VM Malone RR Torres JE Hunsaker LA Piper RC Makler MT Vander Jagt DL Selective inhibitors of human lactate dehydrogenases and lactate dehydrogenase from the malarial parasite *Plasmodium falciparum* J Med Chem 1998 41 3879 3887 10.1021/jm980334n 9748363

[B12] Makler MT Hinrichs DJ Measurement of the lactate dehydrogenase activity of *Plasmodium falciparum *as an assessment of parasitemia Am J Trop Med Hyg 1993 48 205 210 8447524 10.4269/ajtmh.1993.48.205

[B13] Martin SK Rajasekariah GH Awinda G Waitumbi J Kifude C Unified parasite lactate dehydrogenase and histidine-rich protein ELISA for quantification of *Plasmodium falciparum* Am J Trop Med Hyg 2009 80 516 522 19346368

[B14] Cooke AH Chiodini PL Doherty T Moody AH Ries J Pinder M Comparison of a parasite lactate dehydrogenase-based immunochromatographic antigen detection assay (OptiMAL) with microscopy for the detection of malaria parasites in human blood samples Am J Trop Med Hyg 1999 60 173 176 10072131 10.4269/ajtmh.1999.60.173

[B15] Chaikuad A Fairweather V Conners R Joseph-Horne T Turgut-Balik D Brady RL Structure of lactate dehydrogenase from *Plasmodium vivax*: complexes with NADH and APADH Biochemistry 2005 44 16221 16228 10.1021/bi051416y 16331982

[B16] Dunn CR Banfield MJ Barker JJ Higham CW Moreton KM Turgut-Balik D Brady RL Holbrook JJ The structure of lactate dehydrogenase from Plasmodium falciparum reveals a new target for anti-malarial design Nat Struct Biol 1996 3 912 915 10.1038/nsb1196-912 8901865

[B17] Maltha J Gillet P Cnops L van den Ende J van Esbroeck M Jacobs J Malaria rapid diagnostic tests: *Plasmodium falciparum *infections with high parasite densities may generate false positive *Plasmodium vivax *pLDH lines Malar J 2010 9 198 10.1186/1475-2875-9-198 20618990 PMC2911472

[B18] Sabbatani S Fiorino S Manfredi R The emerging of the fifth malaria parasite (Plasmodium knowlesi): a public health concern? Braz J Infect Dis 2010 14 299 309 10.1590/S1413-86702010000300019 20835518

[B19] Hurdayal R Achilonu I Choveaux D Coetzer TH Dean Goldring JP Anti-peptide antibodies differentiate between plasmodial lactate dehydrogenases Peptides 2010 31 525 532 10.1016/j.peptides.2010.01.002 20093160

[B20] Tomar D Biswas S Tripathi V Rao DN Development of diagnostic reagents: raising antibodies against synthetic peptides of PfHRP-2 and LDH using microsphere delivery Immunobiology 2006 211 797 805 10.1016/j.imbio.2006.05.003 17113917

[B21] Alonso PL Barnwell JW Bell D Hanson K Mendis K Moonen B Newman RD de Savigny D Schapira A Slutsker L Tanner M Teuscher T A research agenda for malaria eradication: diagnoses and diagnostics PLoS Med 2010 8 e1000396 10.1371/journal.pmed.1000396 PMC3026696 21311583

[B22] Murray CK Gasser RA Jr Magill AJ Miller RS Update on rapid diagnostic testing for malaria Clin Microbiol Rev 2008 21 97 110 10.1128/CMR.00035-07 18202438 PMC2223842

[B23] Srinivasan S Moody AH Chiodini PL Comparison of blood-film microscopy, the OptiMAL dipstick, Rhodamine-123 fluorescence staining and PCR, for monitoring antimalarial treatment Ann Trop Med Parasitol 2000 94 227 232 10.1080/00034980050006393 10884866

